# Supplementary material for: Ophthalmomyiasis Externa and Importance of Risk Factors, Clinical Manifestations, and Diagnosis: Review of the Medical Literature
Source: Diseases. 2023 Dec 11;11(4):180. doi: 10.3390/diseases11040180 (PMC10743177; doi:10.3390/diseases11040180)
Supplement: Supplementary file 1 [file diseases-11-00180-s001.zip › diseases-2691671-supplementary.pdf]

Supplementary Table S1. General characteristics and clinical pictures of external ophthalmomyiasis cases reported from 2000 to 2022.

Author (year)	Country	Sex	Age	Activity	Cause	Symptoms	Location	Examination	Agent
Weinand et al. 2001 [108]	Germany	M	28		Something strikes the eye (autochthonous)	Foreign body sensation	Left	Conjunctival hyperemia, presence of mobile larvae	*Oestrus ovis*
Emborsky et al. 2002 [113]	USA	M	4			Swelling and papules on lower and upper left eyelid	Left	Presence of three papules and a whitish, eyelid edema and hyperemia, mobile foreign body.	*Dermatobia hominis*
Sigauke et al 2003 [14]	USA	F	16	Student		Pain, mucoid discharge	Both eyes	Conjunctival follicular reaction	No
Denion et al 2004 [17]	Brazil	M	25				Eyelid (right upper)	Furuncular blepharitis	*Dermatobia hominis*
	French Guiana	F	1				Eyelid (right lower)	Edema and pore near the inner canthus	*Dermatobia hominis*
	French Guiana	F	3				Eyelid (left upper and lower)	Movements observed in the dome-shaped lesion.	*Dermatobia hominis*
	French Guiana	M	39	Gold washer		Pain, writhing sensation	Eyelid (right upper)	Serosanguinous and purulent discharge after larval death.	*Dermatobia hominis*
	French Guiana	M	3			Pruritus		Palpebral edema, pore in the conjunctival fornix	*Dermatobia hominis*
	French Guiana	M	13			Stinging sensation, movements within the lesion		Intermittently protruding caruncle larvae	*Dermatobia hominis*
	French Guiana		15				Eyelid (left upper)	Dome-shaped lesion, Pore in free margin	*Dermatobia hominis*
	French Guiana		1				Eyelid (right upper)	Erythema and moderate edema around the pore	*Dermatobia hominis*
	French Guiana	M	45				Eyelid (right upper)	Bloody discharge, Edema, Pore in thickened free margin	*Dermatobia hominis*
Gregory et al. 2004 [58]	Iraq	M	20	US Soldier		Foreign body sensation, watery	Left	Conjunctival congestion, chemosis,	*Oestrus ovis*

						eyes, photophobia		periorbital edema and erythema	
Masoudi et al. 2004 [75]	Iran	M	15			Foreign body sensation, watery eyes, pruritus, eye redness, swelling		Presence of mobile larvae, chemosis	*Oestrus ovis*
	Iran	M	18			Foreign body sensation, watery eyes, pruritus, eye redness, pain, swelling		Presence of mobile larvae, chemosis	*Oestrus ovis*
	Iran	M	24			Foreign body sensation, watery eyes, pain, swelling, chemosis		Presence of mobile larvae, chemosis	*Oestrus ovis*
	Iran	M	27			Foreign body sensation, watery eyes, pruritus, eye redness, swelling		Presence of mobile larvae, chemosis	*Oestrus ovis*
	Iran	M	34			Foreign body sensation, watery eyes, pruritus, eye redness, swelling		Presence of mobile larvae, chemosis	*Oestrus ovis*
	Iran	M	36			Watery eyes, pruritus, eye redness, swelling		Presence of mobile larvae, chemosis, rhinorrhea	*Oestrus ovis*
	Iran	F	41			Foreign body sensation, watery eyes, pruritus, eye redness, swelling		Presence of mobile larvae, chemosis	*Oestrus ovis*
	Iran	M	56			Foreign body sensation, watery eyes, pruritus, eye redness, pain, swelling		Presence of mobile larvae, chemosis	*Oestrus ovis*
Levett et al. 2004 [56]	Barbados	F	55			Foreign body sensation, rhinorrhea, sneezing	Right	Presence of mobile larvae	*Oestrus ovis*
Ashenhurst (2004) [55]	Canada	F	50	Tourist traveled to Belize	Insect bite in the eye	Pain, periorbital erythema, edema of the lower eyelid	Left	Fistulous tract of erythema with periorbital edema around the lower eyelid region.	*Dermatobia hominis*
Dono et al. 2005 [45]	Italy	M			Insect flying around the face	Foreign body sensation,			*Oestrus ovis*

						pruritus, pain, burning eye			
	Italy	M			A sensation of water drop entry	Foreign body sensation, pruritus, pain, burning eye			*Oestrus ovis*
	Italy	M				Foreign body sensation, pruritus, pain, burning eye			*Oestrus ovis*
Huynh et al. 2005 [114]	USA	M	60		History of palpebral trauma	Pain, eye redness, swelling	Right	Upper and lower eyelid edema with erythema and a 1.5 mm round ulceration in the external canthal region.	*Lucilia sericata*
Verstrynge et al. 2005 [57]	Belgium	F	26	Tourist in Greece		Foreign body sensation, watery eyes	Right	Congested conjunctiva, presence of mobile larvae.	*Oestrus ovis*
Miura et al. 2005 [53]	Japan	M	72		Unconscious patient	Presence of larvae	Left	Conjunctival hyperemia, hemorrhage with discharge	*Boettcherisca peregrine*
Beltran et al. 2006 [115]	Peru	M	50	Fisherman	Come from cattle-raising areas	Foreign body sensation, eye redness, eyelid edema, photophobia, decreased visual acuity	Left		*Oestrus ovis*
	Peru	F	67	Laundress	Come from cattle-raising areas	Foreign body sensation, eye redness, eyelid edema, chemosis, decreased visual acuity	Right		*Oestrus ovis*
	Peru	M	11	Student	Come from cattle-raising areas	Foreign body sensation, eye redness, eyelid edema, photophobia	Right		*Oestrus ovis*
Caça et al. 2006 [89]	Turkey	F	8	Farmer's daughter	An ulcerated area on the lateral canthus and zygomatic area	An outgrowth of maggots from the wound around the eye	Right	Lower eyelid and lower palpebral conjunctiva involvement and destruction.	*Chrysomya bezziana*
Ali et al. 2006 [108]	Pakistan	F	49			Pruritus, eye redness, photophobia	Left	Follicular conjunctivitis, hyperemia, mild visual loss.	*Family Oestridae*

	Pakistan	M	19		Get hit by a fly	Foreign body sensation, eye redness		Presence of mobile larvae	*Oestrus ovis*
Wakamatsu et al. 2006 [93]	Brazil	M	11			Pruritus, pain, edema on the inner cantus	Right	Periorbital edema, small erythematous lesion (inner canthus) with a well-defined spot in the center	*Dermatobia hominis*
Bali J et al. 2007 [65]	India	M	50	Manual laborer	Trauma to the eyelid	Swelling, ulceration	Eyelid (right lower)	Presence of maggots, preseptal cellulitis, ulceration, induration, bloody discharge	No
Price et al. 2007 [112]	USA	F	5			Swelling	Eyelid (left upper)	Eyelid edema and hyperemia, presence of small opening with clear discharge.	*Dermatobia hominis*
Misra et al. 2008 [37]	India	F	34	Housewife	Get hit by a fly	Foreign body sensation, watery eyes, eye redness	Right	Conjunctival congestion, presence of mobile larvae, eyelid edema	*Oestrus ovis*
	India	M	4				Left	Presence of a mobile larvae	*Oestrus ovis*
	India	M	40	Farmer	Get hit by a fly		Left		*Oestrus ovis*
	India	F	8	Student			Left		*Oestrus ovis*
	India	F	15	Student	Get hit by a fly		Left		*Oestrus ovis*
	India	M	29	Pastor	Get hit by a fly		Right		*Oestrus ovis*
	India	M	30	Farmer	Get hit by a fly		Right	Presence of mobile larvae	*Oestrus ovis*
	India	M	16	Pastor	Get hit by a fly		Both eyes		*Oestrus ovis*
	India	F	21	Teacher			Left		*Oestrus ovis*
	India	M	26	Farmer	Get hit by a fly		Right		*Oestrus ovis*
	India	F	29	Farmer	Get hit by a fly		Right		*Oestrus ovis*
	India	M	18	Pastor			Left	Presence of mobile larvae	*Oestrus ovis*
	India	F	56	Farmer	Get hit by a fly		Left		*Oestrus ovis*
Dunbar et al. 2008 [102]	Afghanistan	F	29	Nurse	Get hit by a fly	Foreign body sensation, watery eyes, pain, burning eye		Presence of mobile larvae	*Oestrus ovis*
Eyigör et al. 2008 [91]	Turkey	M	33	Research Assistant	Contact with sheep	Mobile foreign body sensation	Right	Presence of mobile larvae	*Oestrus ovis*
Stacey et al. 2008 [106]	Afghanistan	M	18	UK Soldier		Mobile foreign body sensation, irritation	Left	Presence of mobile larvae, follicular reaction and	*Oestrus ovis*

								diffuse injection of the conjunctiva	
Thabit et al. 2008 [88]	Jordan (32)	M 65% (32)	31.6			Foreign body sensation, watery eyes, pruritus, eye redness, swelling	Right (28), Left (21)	Conjunctival hyperemia, presence of mobile larvae, chemosis, watery eyes, mild to severe conjunctivitis, eyelid edema, pruritus, pain	No
	Jordan (17)	F 35% (17)							
Alhady et al. 2008 [54]	Indonesia	M	9		Co-infestation otomiasis-ophthalmomyiasi s	Red eye	Right	A breach in the superonasal bulbar conjunctiva and a live maggot deep in the wound.	*Chrysomya bezziana*
Kemmanu et al. 2009 [29]	India	M	15	Farmer	A fly landed on the eye	Foreign body sensation	Left	Conjunctival congestion, sub-conjunctival hemorrhage, mucoid discharge, punctate keratitis.	*Oestrus ovis*
Thakur et al. 2009 [3]	India	F	17	Student	Exposed to a dust storm	Foreign body sensation, watery eyes, irritation	Right	Congested conjunctiva, presence of small transparent worms	*Oestrus ovis*
Pandey et al. 2009 [63]	India	M	25	Farmer	Something entered the eye while resting under a tree.	Foreign body sensation, watery eyes, burning eye	Right	Congestive conjunctiva, presence of mobile larvae, watery eyes	*Oestrus ovis*
Rivasi et al. 2009 [36]	Italy	M	54	Office Clerk	Foreign body sensation while walking	Watery eyes, photophobia, irritation	Left	Conjunctiva hyperemia, watery exudate, presence of small transparent bodies.	*Oestrus ovis*
Jenzeri et al. 2009 [39]	Tunisia	M	77	Farmer		Foreign body sensation, eye redness, pain, photophobia	Right	Congested conjunctiva, presence of mobile larvae, diffuse stromal corneal edema, various sub-epithelial opacities	*Oestrus ovis*
Cucera et al. 2009 [99]	Germany	M	30	Tourist in Corsica	Contact with flies	Foreign body sensation, watery eyes, eye redness, swelling (eyelids)	Left	Conjunctival hyperemia and congestion, presence of mobile larvae, chemosis, palpebral edema	*Oestrus ovis*
	Germany	F	30	Tourist in Corsica	Contact with flies	Foreign body sensation, watery	Left	Conjunctival hyperemia and congestion,	*Oestrus ovis*

						eyes, eye redness, swelling (eyelids)		presence of mobile larvae, chemosis, palpebral edema	
Kuk et al. 2009 [92]	Turkey	M	40		Get hit by a fly	Foreign body sensation, eye redness	Right	Presence of mobile larvae, chemosis, cornea with punctate keratitis	*Oestrus ovis*
Abuelhssan 2010 [146]	Oman	M	9		Get hit by a fly	Foreign body sensation, watery eyes	Right	Congested conjunctiva, presence of five mobile larvae, watery eyes	*Oestrus ovis*
Anane et al. 2010 [1]	Tunisia	M	6	Student	Get hit by a fly	Foreign body sensation, watery eyes, pruritus, photophobia		Conjunctival hyperemia, presence of mobile larvae	*Oestrus ovis*
	Tunisia	M	30	Technician	Get hit by a fly	Foreign body sensation, watery eyes, pruritus, photophobia		Conjunctival hyperemia, presence of mobile larvae	*Oestrus ovis*
	Tunisia	M	63	Retiree	Get hit by a fly	Foreign body sensation, watery eyes, pruritus, photophobia		Conjunctival hyperemia, presence of mobile larvae	*Oestrus ovis*
	Tunisia	M	20	Worker	Get hit by a fly	Foreign body sensation, watery eyes, pruritus, photophobia		Conjunctival hyperemia, presence of mobile larvae	*Oestrus ovis*
	Tunisia	F	17	Student	Get hit by a fly	Foreign body sensation, watery eyes, pruritus, photophobia		Conjunctival hyperemia, presence of mobile larvae	*Oestrus ovis*
	Tunisia	M	41	Banker	Get hit by a fly	Foreign body sensation, watery eyes, pruritus, photophobia		Conjunctival hyperemia, presence of mobile larvae	*Oestrus ovis*
	Tunisia	M	34	Merchant	Get hit by a fly	Foreign body sensation, watery eyes, pruritus, photophobia		Conjunctival hyperemia, presence of mobile larvae	*Oestrus ovis*
	Tunisia	M	16	Artisan	Get hit by a fly	Foreign body sensation, watery eyes, pruritus, photophobia		Presence of mobile larvae, chemosis	*Oestrus ovis*
	Tunisia	M	8	Student	Get hit by a fly	Foreign body sensation, watery eyes, pruritus, photophobia		Conjunctival hyperemia, presence of mobile larvae	*Oestrus ovis*
	Tunisia	F	45	Housewife	Get hit by a fly	Foreign body sensation, watery eyes, pruritus, photophobia		Presence of mobile larvae, chemosis	*Oestrus ovis*

	Tunisia	M	60	Tourist	Get hit by a fly	Foreign body sensation, watery eyes, pruritus, photophobia		Conjunctival hyperemia, presence of mobile larvae	*Oestrus ovis*
Smillie et al. 2010 [59]	United Kingdom	F	51	Tourist to Morocco		Foreign body sensation, pruritus, eye congestion	Right	Presence of larvae	*Oestrus ovis*
Khurana et al. 2010 [66]	India	F	30			Foreign body sensation, watery eyes	Right	Presence of a mobile larvae	*Oestrus ovis*
	India	F	17		Get hit by a fly	Foreign body sensation, watery eyes	Right	Presence of a mobile larvae	*Oestrus ovis*
Arslan et al. 2010 [144]	Turkey (Istanbul)	M	25	Medical student	Insect flying around the face	Pruritus, eye redness, pain, burning eye	Left	Conjunctival hyperemia, presence of mobile larvae, watery eyes	*Oestrus ovis*
Sreejith et al. 2010 [64]	India	M	35		Dust entered the eye	Watery eyes, eye redness, swelling (eyelid), irritation	Right	Presence of mobile larvae, periorbital and palpebral edema, mucous secretion, conjunctival hemorrhages	*Oestrus ovis*
Abdellatif et al. 2011 [18]	Libya	F			Insect flying around the face	Eye redness		Presence of larvae	*Oestrus ovis*
	Libya	F			Insect flying around the face	Eye redness		Presence of larvae	*Oestrus ovis*
	Libya	F			Insect flying around the face	Eye redness		Presence of larvae	*Oestrus ovis*
	Libya	F			Insect flying around the face	Eye redness		Presence of larvae	*Oestrus ovis*
	Libya	F			Insect flying around the face	Eye redness		Presence of larvae	*Oestrus ovis*
	Libya	F			Insect flying around the face	Eye redness		Presence of larvae	*Oestrus ovis*
	Libya	F			Insect flying around the face	Eye redness		Presence of larvae	*Oestrus ovis*
	Libya	M			Insect flying around the face	Eye redness		Presence of larvae	*Oestrus ovis*
	Libya	M			Insect flying around the face	Eye redness		Presence of larvae	*Oestrus ovis*
	Libya	M			Insect flying around the face	Eye redness		Presence of larvae	*Oestrus ovis*
	Libya	M			Insect flying around the face	Eye redness		Presence of larvae	*Oestrus ovis*
	Libya	M			Insect flying around the face	Eye redness		Presence of larvae	*Oestrus ovis*
	Libya	M			Insect flying around the face	Eye redness		Presence of larvae	*Oestrus ovis*

	Libya	M			Insect flying around the face	Eye redness		Presence of larvae	*Oestrus ovis*
	Libya	M			Insect flying around the face	Eye redness		Presence of larvae	*Oestrus ovis*
	Libya	M			Insect flying around the face	Eye redness		Presence of larvae	*Oestrus ovis*
	Libya	M			Insect flying around the face	Eye redness		Presence of larvae	*Oestrus ovis*
	Libya	M			Insect flying around the face	Eye redness		Presence of larvae	*Oestrus ovis*
	Libya	M			Insect flying around the face	Eye redness		Presence of larvae	*Oestrus ovis*
	Libya	M			Insect flying around the face	Eye redness		Presence of larvae	*Oestrus ovis*
	Libya	M			Insect flying around the face	Eye redness		Presence of larvae	*Oestrus ovis*
Yar et al. 2011 [90]	Turkey	F	43			Foreign body sensation, pruritus, eye redness, pain	Right	Conjunctival hyperemia, presence of mobile larvae, eyelid edema and hyperemia (right)	*Oestrus ovis*
	Turkey	M	21			Eye redness, eye burning	Right	Conjunctival hyperemia, presence of mobile larvae	*Oestrus ovis*
Ramirez-Miranda et al. 2011 [19]	Mexico	M	40		Something hit in the face	Foreign body sensation, watery eyes, pruritus, eye redness	Both eyes	Presence of a mobile larvae	*Oestrus ovis*
Chakraborti et al. 2011 [67]	India	M	35	Merchant	Living with animals	Pruritus, burning eye	Left	Conjunctival congestion, presence of mobile larvae	*Oestrus ovis*
	India	M	24	Shoemaker	Living with animals	Watery eyes, eye redness, irritation	Left	Conjunctival congestion, presence of mobile larvae	*Oestrus ovis*
Singh et al. 2012 [27]	India	M	11			Foreign body sensation, watery eyes, pruritus	Both eyes	Conjunctival hyperemia, presence of mobile larvae, chemosis, palpebral edema	*Oestrus ovis*
	India	M	45	Farmer		Foreign body sensation, watery eyes, pruritus	Right	Conjunctival hyperemia, presence of mobile larvae, chemosis, palpebral edema	*Oestrus ovis*
	India	M	25	Farmer		Foreign body sensation, watery eyes, pruritus	Right	Conjunctival hyperemia, presence of mobile	*Oestrus ovis*

								larvae, chemosis, palpebral edema	
Shankar et al. 2012 [62]	India	M	50	Farmer	Something entered the eye while working	Foreign body sensation, watery eyes, eye redness, pain	Left	Congested conjunctiva, presence of mobile larvae, watery eyes	*Oestrus ovis*
Vijayalekshmi et al. 2013 [79]	India	F	25	Veterinary	Something entered the eye	Foreign body sensation, watery eyes, burning eye	Left	Congested conjunctiva, watery eyes	*Oestrus ovis*
Akdemir et al. 2013 [26]	Turkey	F	18			Foreign body sensation, watery eyes, eyelid edema.		Conjunctival hyperemia, chemosis, eyelid edema, sub-conjunctival hemorrhage.	*Oestrus ovis*
	Turkey	F	23		Exposure to flies	Foreign body sensation, watery eyes, eye redness		Conjunctival hyperemia	*Oestrus ovis*
	Turkey	M	12			Foreign body sensation, watery eyes, eye redness		Conjunctival hyperemia, chemosis	*Oestrus ovis*
	Turkey	F	45		Exposure to flies	Foreign body sensation, watery eyes, eye redness		Conjunctival hyperemia, chemosis	*Oestrus ovis*
	Turkey	M	61		Exposure to flies	Foreign body sensation, watery eyes, eye redness, eyelid edema		Conjunctival hyperemia, chemosis, palpebral edema.	*Oestrus ovis*
	Turkey	M	28		Exposure to flies	Foreign body sensation, watery eyes, eye redness, eyelid edema		Conjunctival hyperemia, chemosis, palpebral edema, punctate epithelial erosions.	*Oestrus ovis*
	Turkey	M	17			Foreign body sensation, watery eyes, eye redness		Conjunctival hyperemia, chemosis	*Oestrus ovis*
	Turkey	M	13			Foreign body sensation, watery eyes, eye redness		Conjunctival hyperemia, chemosis	*Oestrus ovis*
	Turkey	F	15		Exposure to flies	Foreign body sensation, watery eyes, eye redness, eyelid edema		Conjunctival hyperemia, chemosis, palpebral edema, punctate epithelial erosions	*Oestrus ovis*
	Turkey	F	48		Exposure to flies	Foreign body sensation, watery eyes, eye redness, eyelid edema		Conjunctival hyperemia, chemosis, eyelid edema.	*Oestrus ovis*

Sucilathangam et al. 2013 [38]	India	M	25	Farmer		Foreign body sensation, watery eyes, pruritus		Conjunctival hyperemia, watery eyes, eyelid edema, chemosis, rhinorrhea	*Oestrus ovis*
	India	M	28	Farmer		Foreign body sensation, watery eyes, pruritus, pain		Conjunctival hyperemia, chemosis, eyelid edema, watery eyes	*Oestrus ovis*
	India	M	42	Farmer		Foreign body sensation, pruritus, pain, burning eye		Chemosis, watery eyes, eyelid edema	*Oestrus ovis*
	India	M	47	Farmer		Foreign body sensation, watery eyes, pruritus, burning eye		Conjunctival hyperemia, chemosis, watery eyes, palpebral edema	*Oestrus ovis*
	India	M	35	Farmer		Foreign body sensation, watery eyes, pruritus		Conjunctival hyperemia, chemosis, watery eyes, palpebral edema	*Oestrus ovis*
	India	M	25	Farmer		Foreign body sensation, watery eyes, pruritus, burning eye		Conjunctival hyperemia, chemosis, eyelid edema, rhinorrhea	*Oestrus ovis*
	India	F	30	Farmer		Foreign body sensation, watery eyes, pruritus, burning eye		Conjunctival hyperemia, chemosis, watery eyes, palpebral edema	*Oestrus ovis*
	India	M	65	Farmer		Foreign body sensation, watery eyes, pruritus, pain, burning eye		Conjunctival hyperemia, chemosis, watery eyes, palpebral edema	*Oestrus ovis*
	India	M	35	Farmer		Foreign body sensation, watery eyes, pruritus, pain, burning eye		Conjunctival hyperemia, watery eyes, eyelid edema, chemosis, rhinorrhea	*Oestrus ovis*
	India	F	26	Farmer		Foreign body sensation, pruritus, pain, burning eye		Conjunctival hyperemia, chemosis, watery eyes, eyelid edema	*Oestrus ovis*
Graffi et al. 2013 [128]	Israel	M	91	Elderly handicapped	Patient hospitalized with underlying disease		Both eyes	Congested conjunctiva, presence of a mobile larvae in both cul-de-sacs,	*Sarcophaga argyrostoma*

								mucopurulent discharge.	
Taormina et al. 2013 [111]	USA	F	1			Eye redness, swelling in the periorbital region	Left	Eyelid edema, erythema, and edema (upper and lower) with a punctate sinus tract in the upper eyelid draining serosanguinous material.	*Dermatobi hominis*
Tomy et al. 2013 [13]	India	M	70	Elderly with underlying disease	Involvement of periorbital tissues due to malignant disease	Presence of foul-smelling ulcers in the wall of the eye, riddled with maggots.	Right	Eyelids thickened and inflamed, ulcerative cavities filled with maggots, exposure keratitis.	*Musca domestica*
Choudhary et al. 2013 [77]	India	F	16		Ram piece injury.	Foreign body sensation, watery eyes, mild conjunctivitis		Presence of larvae, conjunctivitis	*Oestrus ovis*
	India	F	14		Get hit by a fly	Foreign body sensation, watery eyes, pruritus		Presence of larvae, conjunctivitis (mild) with mucoid discharge	*Oestrus ovis*
	India	F	18	Peasant	Foreign body sensation while working	Foreign body sensation, watery eyes, eye redness		Presence of larvae, conjunctivitis (mild)	*Oestrus ovis*
	India	M	65		Get hit by a fly	Watery eyes, eye redness, pain, mucopurulent discharge		Presence of larvae, chemosis, congestion, punctate sub-conjunctival hemorrhages	*Oestrus ovis*
	India	F	16		Get hit by a fly	Foreign body sensation, eye redness, pain		Presence of larvae, conjunctivitis (mild)	*Oestrus ovis*
	India	F	25	Peasant	Foreign body sensation while working	Foreign body sensation, watery eyes, eye redness		Presence of larvae, conjunctivitis	*Oestrus ovis*
	India	F	18		Get hit by a fly	Watery eyes, eye redness, pain		Presence of larvae, conjunctivitis	*Oestrus ovis*
Carrillo et al. 2013 [109]	Spain	M	26	Hiker	Get hit by a fly	Foreign body sensation	Left	Presence of larvae	*Oestrus ovis*
	Spain	M	34		Get hit by a fly	Watery eyes, pruritus	Right	Presence of larvae in the conjunctival fornix	*Oestrus ovis*
	Spain	M	22			Mobile foreign body sensation, eye redness	Left	Presence of larvae in the conjunctival fornix	*Oestrus ovis*
Pather et al. 2013 [51]	South Africa	M	30	Construction worker		Eye redness, pain	Left	Conjunctival hyperemia,	*Oestrus ovis*

						presence of mobile larvae, chemosis	
Gholamhossein et al. 2013 [40]	Iran	M	Farmer living in a rural region	Travel to desert regions or living with sheep	Foreign body sensation, eye redness, eyelid edema	Conjunctival hyperemia, palpebral edema	*Oestrus ovis*
	Iran	M	Farmer living in a rural region	Travel to desert regions or living with sheep	Foreign body sensation, eye redness, eyelid edema	Conjunctival hyperemia, palpebral edema	*Oestrus ovis*
	Iran	M	Farmer living in a rural region	Travel to desert regions or living with sheep	Foreign body sensation, eye redness, eyelid edema	Conjunctival hyperemia, palpebral edema	*Oestrus ovis*
	Iran	M	Farmer living in a rural region	Travel to desert regions or living with sheep	Foreign body sensation, eye redness, eyelid edema	Conjunctival hyperemia, chemosis, a viscous pattern of discharge	*Oestrus ovis*
	Iran	M	Farmer living in a rural region	Travel to desert regions or living with sheep	Foreign body sensation, eye redness, eyelid edema	Conjunctival hyperemia, chemosis, a viscous pattern of discharge	*Oestrus ovis*
	Iran	M	Farmer living in a rural region	Travel to desert regions or living with sheep	Foreign body sensation, eye redness, eyelid edema	Conjunctival hyperemia, chemosis, a viscous pattern of discharge	*Oestrus ovis*
	Iran	M	Farmer living in a rural region	Travel to desert regions or living with sheep	Foreign body sensation, eye redness, eyelid edema	Conjunctival hyperemia, chemosis, a viscous pattern of discharge	*Oestrus ovis*
	Iran	M	Farmer living in a rural region	Travel to desert regions or living with sheep	Foreign body sensation, eye redness, eyelid edema	Conjunctival hyperemia, chemosis, a viscous pattern of discharge	*Oestrus ovis*
	Iran	M	Farmer living in a rural region	Travel to desert regions or living with sheep	Foreign body sensation, eye redness, eyelid edema	Conjunctival hyperemia, chemosis, a viscous pattern of discharge	*Oestrus ovis*
	Iran	M	Farmer living in a rural region	Travel to desert regions or living with sheep	Foreign body sensation, eye redness, eyelid edema	Conjunctival hyperemia, chemosis, a viscous pattern of discharge	*Oestrus ovis*
	Iran	M	Farmer living in a rural region	Travel to desert regions or living with sheep	Foreign body sensation, eye	Conjunctival hyperemia, chemosis, a viscous	*Oestrus ovis*

						redness, eyelid edema		pattern of discharge	
	Iran	M		Farmer living in a rural region	Travel to desert regions or living with sheep	Foreign body sensation, eye redness, eyelid edema		Conjunctival hyperemia, chemosis, a viscous pattern of discharge	*Oestrus ovis*
	Iran	M		Farmer living in a rural region	Travel to desert regions or living with sheep	Foreign body sensation, eye redness, eyelid edema		Conjunctival hyperemia, chemosis, a viscous pattern of discharge	*Oestrus ovis*
	Iran	M		Farmer living in a rural region	Travel to desert regions or living with sheep	Foreign body sensation, eye redness, eyelid edema		Conjunctival hyperemia, chemosis, a viscous pattern of discharge	*Oestrus ovis*
	Iran	M		Farmer living in a rural region	Travel to desert regions or living with sheep	Foreign body sensation, eye redness, eyelid edema		Conjunctival hyperemia, chemosis, a viscous pattern of discharge	*Oestrus ovis*
	Iran	M		Farmer living in a rural region	Travel to desert regions or living with sheep	Foreign body sensation, eye redness, eyelid edema		Conjunctival hyperemia, chemosis, a viscous pattern of discharge	*Oestrus ovis*
	Iran	M		Farmer living in a rural region	Travel to desert regions or living with sheep	Foreign body sensation, eye redness, eyelid edema		Conjunctival hyperemia, chemosis, a viscous pattern of discharge	*Oestrus ovis*
	Iran	M		Farmer living in a rural region	Travel to desert regions or living with sheep	Foreign body sensation, eye redness, eyelid edema		Conjunctival hyperemia, chemosis, a viscous pattern of discharge	*Oestrus ovis*
Neimer et al. 2014 [31]	Israel	M	27		Get hit by an insect	Watery eyes, pain, eye burning	Left	Hyperemia, swollen and erythematous palpebral, watery eyes	*Oestrus ovis*
	Israel	M	17		Get hit by a fly (face)	Mobile foreign body sensation, irritation	Left	Hyperemia, swollen and erythematous palpebral, watery eyes	*Oestrus ovis*
Ranjan et al. 2014 [34]	Oman	M	72	Homeless person	Trauma to the eyelid	Pain, ulceration, discomfort	Eyelid (left upper)	Ulceration, erythema, and edema of the periorbital tissues, necrosis upper	No

								eyelid with numerous maggots.	
Istek 2014 [68]	Turkey	M	30	Farmer	Contact with sheep	Foreign body sensation	Right	Conjunctival hyperemia, presence of mobile larvae, serous discharge	*Oestrus ovis*
	Turkey	F	26		A fly landed on the eye	Mobile foreign body sensation	Right	Conjunctival hyperemia, presence of mobile larvae, mucopurulent discharge, palpebral cellulitis	*Oestrus ovis*
	Turkey	M	10	Farmer	A fly landed on the eye	Red eye		Presence of mobile larvae in the lacrimal meniscus	*Oestrus ovis*
Kalezic et al. 2014 [158]	Serbia	F	87	Disabled elderly woman	Severe neurological damage with predisposing factors		Left	Presence of larvae on the conjunctival surface	*Lucilia sericata*
Akçakaya et al. 2014 [25]	Turkey	M	17	Student	A fly landed on the eye	Mobile foreign body sensation, watery eyes, pain, irritation	Left	Conjunctival hyperemia, presence of mobile larvae, eyelid edema (left)	*Oestrus ovis*
Calışkan et al. 2014 [28]	Turkey	M	17			Watery eyes, pruritus, eye redness	Both eyes	Conjunctival hyperemia, presence of mobile larvae, eyelid edema (bilateral)	*Oestrus ovis*
	Turkey	F	25		Get hit by a fly	Foreign body sensation, watery eyes	Both eyes	Conjunctival hyperemia, presence of mobile larvae, bilateral conjunctival congestion	*Oestrus ovis*
	Turkey	M	31	Merchant	Get hit by a fly	Foreign body sensation, watery eyes	Both eyes	Presence of mobile larva, chemosis (bilateral)	*Oestrus ovis*
Al-Amry et al. 2014 [147]	Saudi Arabia	M	50		Went through a sheep farming area	Mobile foreign body sensation, watery eyes, eye redness	Right	Conjunctival chemosis, presence of mobile larvae	*Oestrus ovis*
Garcia-Guerrero et al. 2014 [20]	Mexico	F	16	Student	Get hit by an insect	Foreign body sensation, eye redness, pain	Left	Congested conjunctivitis, presence of mobile larvae	*Oestrus ovis*

Murga 2014 [61]	Bolivia	F	35		Something entered the eye	Mobile foreign body sensation, pruritus	Left	Presence of mobile larvae	*Oestrus ovis*
Alameri 2014 [42]	Jordan	M	25	Farmer	Foreign body sensation	Foreign body sensation, pain, irritation	Right	Presence of mobile larvae, watery eyes, palpebral edema and hyperemia	*Oestrus ovis*
Bonzon et al. 2015 [110]	France	F	45	Teacher	Get hit by a fly	Pruritus, irritation	Right	Presence of larvae	*Oestrus ovis*
	France	F	67	Farmer	Get hit by an insect	Pain			*Oestrus ovis*
	France	F	43	Nurse	Trauma to the eye (fly)	Foreign body sensation			*Oestrus ovis*
	France	M	28	Mason	Trauma to the eye.	Pain	Left		*Oestrus ovis*
Dokur et al. 2015 [72]	Turkey	M	57			Pruritus, eye redness	Left	Conjunctival hyperemia, presence of mobile larvae	*Oestrus ovis*
Choi et al. 2015 [24]	South Korea	M	72	Farmer	No predisposing factors	Foreign body sensation, watery eyes, eye redness, pain	Left	Left periorbital edema, erythema of the left eyelid, and small larvae in the conjunctival pouch.	*Lucilia sericata*
Berrozpe-Villabona et al. 2015 [137]	Spain	M	50	Tourist	Went on vacation to a rural area	Mobile foreign body sensation, watery eyes, pruritus	Left	Presence of mobile larvae, watery eyes, congested bulbar conjunctiva	*Oestrus ovis*
Sundu et al. 2015 [69]	Turkey	M	13		Foreign body hit the eye while passing by a flock of sheep	Watery eyes, pruritus, eyelid edema	Left	Presence of mobile larvae in the palpebral conjunctiva	*Oestrus ovis*
	Turkey	M	21		Foreign body hit the eye	Watery eyes, eye redness, blurred vision	Right	Presence of mobile larvae in the palpebral and bulbar conjunctivae.	*Oestrus ovis*
	Turkey	M	55	Pastor	Larvae removed from the eye (a week earlier).	Watery eyes, eye redness	Right	Presence of mobile larvae in the palpebral and bulbar conjunctivae.	*Oestrus ovis*
Santé Fernandez et al. 2015 [148]	Spain (Canary Islands)	F	43		Contact with flies	Foreign body sensation, eye redness, pain	Right	Presence of a mobile larvae	*Oestrus ovis*
Naimer 2015 [32]	Israel	M	27		Contact with flies	Watery eyes, pain	Left	Presence of larvae, watery eyes, eyelid edema and hyperemia	*Oestrus ovis*

Nene et al. 2015 [87]	India	F	42	Poverty and chronic alcoholism	A wound in the inner canthus	Pruritus, edema, foul-smelling discharge, presence of worms	Right	Presence of mobile larvae, periorbital edema, conjunctival vascular ingurgitation, inner canthal lesion	*Chrysomya bezziana*
Usta et al. 2015 [43]	Turkey	F	4		Dust entered the eye	Watery eyes, pruritus, eye redness, irritation	Right	Conjunctival hyperemia, presence of mobile larvae, watery eyes, eyelid edema	*Oestrus ovis*
	Turkey	F	50	Pastor	Get hit by a fly	Watery eyes, pruritus, eye redness, irritation	Right	Conjunctival hyperemia, presence of mobile larvae, watery eyes, eyelid edema	*Oestrus ovis*
	Turkey	F	45	Farmer	Get hit by a fly	Watery eyes, pruritus, eye redness, irritation	Left	Conjunctival hyperemia, presence of mobile larvae, watery eyes, eyelid edema	*Oestrus ovis*
	Turkey	F	49	Pastor	Get hit by a fly	Watery eyes, pruritus, eye redness, irritation	Right	Conjunctival hyperemia, presence of mobile larvae, watery eyes, eyelid edema	*Oestrus ovis*
	Turkey	F	60	Farmer	Get hit by a fly	Watery eyes, pruritus, eye redness, irritation	Right	Conjunctival hyperemia, presence of mobile larvae, watery eyes, eyelid edema	*Oestrus ovis*
	Turkey	M	18	Student	Get hit by something	Watery eyes, pruritus, eye redness, irritation	Left	Conjunctival hyperemia, presence of mobile larvae, watery eyes, eyelid edema	*Oestrus ovis*
	Turkey	M	41	Worker	Get hit by something	Watery eyes, pruritus, eye redness, irritation	Right	Conjunctival hyperemia, presence of mobile larvae, watery eyes, eyelid edema	*Oestrus ovis*
	Turkey	M	19	Student	Get hit by something	Watery eyes, pruritus, eye redness, irritation	Right	Conjunctival hyperemia, presence of mobile larvae, watery eyes, eyelid edema	*Oestrus ovis*
	Turkey	M	17	Student	Get hit by a fly	Watery eyes, pruritus, eye redness, irritation	Right	Conjunctival hyperemia, presence of mobile larvae, watery eyes, eyelid edema	*Oestrus ovis*

	Turkey	F	33	Housewife	Get hit by something	Watery eyes, pruritus, eye redness, irritation	Left	Conjunctival hyperemia, presence of mobile larvae, watery eyes, eyelid edema	*Oestrus ovis*
	Turkey	M	21	Student	Get hit by a fly	Watery eyes, pruritus, eye redness, irritation	Left	Conjunctival hyperemia, presence of mobile larvae, watery eyes, eyelid edema	*Oestrus ovis*
	Turkey	M	52	Shopkeeper	Get hit by a fly	Watery eyes, pruritus, eye redness, irritation	Right	Conjunctival hyperemia, presence of mobile larvae, watery eyes, eyelid edema	*Oestrus ovis*
Vogt et al. 2015 [149]	Germany	M	26	Tourist to Croatia		Foreign body sensation, pruritus	Left	Conjunctival hyperemia and congestion, presence of mobile larvae, chemosis	*Oestrus ovis*
Pal et al. 2016 [30]	India	F	36		Dust entered the eye	Foreign body sensation, watery eyes, eye redness	Left	Bulbar and palpebral conjunctiva hyperemia and tiny mobile white bodies.	*Oestrus ovis*
Alsaif et al. 2016 [130]	USA	F	10		Visit a farm	Swelling of the eyelid	Eyelid (right upper and lower)	Eyelid edema (right upper and lower), periorbital edema, conjunctival chemosis, cutaneous opening located adjacent to the lateral canthus.	*Dermatobia hominis*
Özyol et al. 2016 [70]	Turkey	F	45		While cutting fruit from a tree	Foreign body sensation, eye redness, pain	Right	Conjunctival hyperemia, presence of mobile larvae, mucopurulent discharge in the cul-de-sacs	*Oestrus ovis*
	Turkey	F	37		Get hit by a fly	Foreign body sensation, watery eyes, eye redness	Right	Conjunctival hyperemia, presence of mobile larvae, eyelid edema, punctate keratitis	*Oestrus ovis*
	Turkey	M	17			Eye redness, pain	Right	Conjunctival hyperemia, presence of mobile larvae, small	*Oestrus ovis*

								conjunctival hemorrhages	
Albaroudi et al. 2016 [52]	Morocco	M	28		Contact with flies	Foreign body sensation, watery eyes, pruritus, eye redness		Conjunctival hyperemia, presence of mobile larvae	*Oestrus ovis*
Rizvi et al. 2016 [86]	India	M	60	Homeless person	History of right eye evisceration one year before.	Painful swelling around the eyes, scalp with discharge, burning eye	Both eyes	Left eye only with senile cataract	*Musca domestica*
Norouzi et al. 2017 [21]	Iran	M	78	Farmer	Previous retinal surgery with predisposing factors	Foreign body sensation, pruritus, pain, eye discharge, presence of worms	Right	Conjunctival hyperemia and congestion, presence of mobile larvae, moderate discharge, palpebral edema	*Lucilia sericata*
Sen et al. 2017 [129]	India	F	52	Farmer	Something entered the eye	Watery eyes, pain, eye burning	Right	Congested conjunctiva, presence of mobile larvae, watery eyes	*Oestrus ovis*
	India	M	75	Farmer	Soil particles hit both eyes	Watery eyes, eye redness, pain	Both eyes	Congested conjunctiva, presence of mobile larvae, watery eyes	*Oestrus ovis*
Villeda et al. 2017 [60]	Honduras	F	8			Foreign body sensation, eyelid edema, conjunctival hyperemia, yellowish discharge	Left	Conjunctival hyperemia, presence of mobile larvae, biparpebral edema, purulent discharge	No
Jenkins et al. 2018 [48]	Australia	M	20	Tourist		Watery eyes, pain, photophobia		Conjunctival hyperemia, presence of three larvae, corneal abrasion	*Oestrus ovis*
Gupta et al. 2018 [80]	India	M	38	Farmer	Exposure to manure	Foreign body sensation	Left	Congested conjunctivae, palpebral edema	*Musca spp.*
Basmaciyan et al. 2018 [117]	France	M	19	Farmer	Trauma to the eye (fly)	Mobile foreign body sensation, pain	Right	Conjunctival hyperemia and congestion, presence of mobile larvae	*Oestrus ovis*
Velev et al. 2018 [47]	Bulgaria	F	61		Get hit by an insect	Foreign body sensation, watery eyes, conjunctival hyperemia	Left	Conjunctival hemorrhage, presence of three mobile larvae, eyelid edema (lower)	*Oestrus ovis*

Zhang et al. 2018 [49]	China	M	30	Vehicle Inspector	Foreign body sensation while closing the door of a farm vehicle.	Foreign body sensation, watery eyes, pruritus	Right	Conjunctival hyperemia, presence of mobile larvae in the tarsal and bulbar conjunctiva., petechial hemorrhages	*Oestrus ovis*
Sharma 2018 [150]	Saudi Arabia	M	32		Exposed to a dust storm	Foreign body sensation, watery eyes, eye redness, rhinorrhea	Left	Congested conjunctiva, presence of larvae, eyelid edema, photophobia, reduced palpebral aperture	*Oestrus ovis*
Rao et al. 2018 [84]	India	M	38		Dust entered the eye	Foreign body sensation, watery eyes, eye redness, pain	Left	Congested conjunctiva, presence of mobile larvae, watery eyes	*Oestrus ovis*
Serra Moltó et al. 2018 [120]	Spain	F	46		From Honduras	Pain, eyelid swelling (upper)	Right	Hyperemia of the upper eyelid, solution of continuity of the tarsal conjunctiva, and extruction of part of a worm.	*Dermatobia hominis*
Tabuenca-del Barrio et al. 2018 [44]	Spain	F	39	Peasant	Get hit by a fly	Foreign body sensation	Right	Presence of mobile larvae	*Oestrus ovis*
	Spain	M	57	Farmer		Foreign body sensation	Right	Presence of mobile larvae	*Oestrus ovis*
	Spain	M	20		Get hit by a fly	Foreign body sensation	Right	Presence of mobile larvae	*Oestrus ovis*
	Spain	M	39	Farmer		Foreign body sensation	Right	Presence of mobile larvae in the conjunctival sac fundus.	*Oestrus ovis*
Couto Junior et al. 2018 [94]	Brazil	M	44			Eyelid edema, irritation, discharge secretion	Right	Presence of mobile larvae, drainage hole in the upper palpebral angle	*Dermatobia hominis*
Armas-Herrera et al. 2018 [151]	Peru	M	14		Lives in a rural area	Foreign body sensation, pruritus, eye redness, irritation	Left	Conjunctival hyperemia, presence of mobile larvae, chemosis	*Oestrus ovis*
Ibáñez-Navarro et al. 2018 [152]	Spain	M	56	Farmer		Foreign body sensation, pruritus	Right	Presence of mobile larvae	No
Fries et al. 2018 [153]	Germany	F	42	Tourist to Greece	Contact with sheep	Mobile foreign body sensation, pruritus	Left	Presence of a mobile larva	*Oestrus ovis*

Sudhir et al. 2018 [154]	India	F	28	Student	Direct splash	Foreign body sensation, watery eyes, pruritus, pain	Left	Multiple larvae in the upper and lower cul-de-sacs	*Oestrus ovis*
	India	M	24	Student	Insect entered the eye	Watery eyes, pruritus, photophobia, irritation	Left	Multiple larvae in the upper and lower cul-de-sacs	*Oestrus ovis*
Gautam et al. 2019 [81]	India	F	26	Peasant	Something entered the eye	Foreign body sensation, watery eyes, pain, irritation	Right	Congested conjunctiva, presence of mobile larvae in the palpebral and bulbar conjunctiva, watery eyes	*Oestrus ovis*
Jordan et al. 2019 [50]	Jamaica	M	17	Farmer	Foreign body sensation	Foreign body sensation, pruritus, eye redness, pain	Left	Conjunctival congestion, presence of three mobile larvae	*Oestrus ovis*
D'Assumpcao et al. 2019 [96]	USA	M	16	Teenager	History of playing on a field fertilized with manure	Mobile foreign body sensation, eye redness, irritation	Left	Conjunctival hyperemia, presence of multiple larvae in bulbar conjunctiva and fornix.	*Oestrus ovis*
Dutta Majumder et al. 2019 [85]	India	M	58			Eye redness, irritation	Right	Presence of mobile larvae, hyperemia of the bulbar and palpebral conjunctiva	*Oestrus ovis*
Mamani-Quispe et al. 2019 [97]	Peru	M	26	Chef		Foreign body sensation, watery eyes, eye redness	Left	Presence of mobile larvae, Mild bulbar conjunctival hyperemia	*Oestrus ovis*
Can et al. 2020 [71]	Turkey	F	55	Cattleman		Pruritus, eye redness, burning pain	Right	Conjunctival hyperemia, eyelids edema	No
Gupta et al. 2020 [16]	India	M	52	Hairdresser	Something entered the eye	Foreign body sensation, eye redness	Left	Conjunctival congestion, chemosis, watery eyes, palpebral edema	*Oestrus ovis*
Hartmannová et al. 2020 [46]	Czech Republic	M		Tourist in Greece	Sawdust entered the eye	Watery eyes, pain	Left	Presence of larvae, minor erosion of the corneal epithelium, hyper-perfusion of the conjunctiva	*Oestrus ovis*
Farias et al. 2020 [11]	Brazil	M	51	Alcoholic	Trauma	Pain, erythema, sensation of worms moving in the eyelid	Eyelid (left lower)	An ulcerated lesion with erythematous margins containing numerous larvae	*Cochliomyia macellaria*

Dadaci et al. 2020 [73]	Turkey	M	47		Attack of a fly while in the garden	Mobile foreign body sensation, pruritus, eye redness	Right	Conjunctival hyperemia, presence of larvae, eyelid edema (upper)	*Oestrus ovis*
Sen et al. 2020 [82]	India	M	1		Cohabitation with domestic animals	Eye redness, edema, ocular discharge, bleeding	Eyelid (right upper and lower)	Conjunctival congestion, presence of mobile larvae, chemosis, periorbital eyelids edema and hyperemia, hyperemic, tender, and edematous skin in the adjacent facial area.	*Oestrus ovis*
Balamurugan et al. 2020 [83]	India	F	26	Farmer	Foreign body entered the eye	Watery eyes, eye redness, mucopurulent discharge	Left	Presence of larvae	*Oestrus ovis*
Pupić-Bakrač et al. 2020 [104]	Republic of Croatia	M	30	Pastor	Get hit by a fly	Foreign body sensation, eye redness, irritation	Right	Conjunctivitis (severe), presence of mobile larvae, chemosis	*Oestrus ovis*
	Republic of Croatia	F	76	Pastor	Get hit by a fly	Eye redness, irritation	Right	Congested conjunctiva, presence of a mobile larva, eyelid edema	*Oestrus ovis*
Kunduracı et al. 2020 [74]	Turkey	F	18			Foreign body sensation, watery eyes, eye redness	Left	Multiple mobile larvae, edema and hyperemia of the left eyelid, chemosis,	*Oestrus ovis*
Jiang et al. 2020 [145]	USA	M	44	Homeless person	Multiple predisposing factors	Pain, headache, blurred vision	Right	Presence of a mobile larva, chemosis, conjunctivitis, central corneal epithelial defect	No
Maharjan et al. 2021 [155]	Nepal	M	22			Foreign body sensation, watery eyes, eye redness, photophobia	Left	Congestive conjunctiva, numerous larvae, watery eyes, eyelid edema	*Oestrus ovis*
	Nepal	F	38		Insect bite in the eye	Foreign body sensation, redness, eyelid edema	Right	Congestive conjunctiva, numerous larvae, watery eyes, eyelid edema	*Oestrus ovis*

Singh et al. 2022 [78]	India	M	45		Foreign body sensation	Mobile foreign body sensation, irritation	Right	Conjunctival congestion, presence of larvae, watery discharge	*Oestrus ovis*
	India	M	32		Foreign body sensation	Foreign body sensation, watery eyes, pain	Right	Conjunctival congestion, presence of larvae, watery discharge	*Oestrus ovis*
	India	M	28		Something strikes the eye	Foreign body sensation, watery eyes, pain	Left	Conjunctival congestion, presence of larvae, watery discharge	*Oestrus ovis*
Abihaidar et al. 2022 [156]	France	M	53		Lived near a horse and sheep farm	Foreign body sensation	Right	Conjunctival hyperemia, presence of mobile larvae	*Oestrus ovis*
Naujokaitis et al. 2022 [157]	Germany	F	42		Contact with flies	Foreign body sensation, eye redness, burning eye	Right	Presence of mobile larvae, conjunctival injection, scanty secretion	*Oestrus ovis*
Tamponi et al. 2022 [98]	Italy	F	30	Tourist	Foreign body entered the eye	Watery eyes, presence of larvae		Conjunctival hyperemia, presence of mobile larvae, blepharospasm	*Oestrus ovis*
	Italy	F	32	Tourist		Mobile foreign body sensation, watery eyes, pruritus, eye redness		Hyperemic conjunctiva, numerous larvae, palpebral edema	*Oestrus ovis*
	Italy	F	17	Tourist	Direct splash	Foreign body sensation, eye burning	Left	Presence of a mobile larva, moderate conjunctival hyperemia	*Oestrus ovis*
